# Dynamic Shannon Performance in a Multiobjective Particle Swarm Optimization

**DOI:** 10.3390/e21090827

**Published:** 2019-08-23

**Authors:** E. J. Solteiro Pires, J. A. Tenreiro Machado, P. B. de Moura Oliveira

**Affiliations:** 1 INESC TEC—INESC Technology and Science (UTAD pole), ECT–UTAD Escola de Ciências e Tecnologia, Universidade de Trás-os-Montes e Alto Douro, 5000-811 Vila Real, Portugal; 2 Department of Electrical Engineering, ISEP—Institute of Engineering, Polytechnic of Porto, Rua Dr. António Bernadino de Almeida, 4249-015 Porto, Portugal

**Keywords:** multiobjective particle swarm optimization, Shannon entropy, solution diversity, front level heterogeneity

## Abstract

Particle swarm optimization (PSO) is a search algorithm inspired by the collective behavior of flocking birds and fishes. This algorithm is widely adopted for solving optimization problems involving one objective. The evaluation of the PSO progress is usually measured by the fitness of the best particle and the average fitness of the particles. When several objectives are considered, the PSO may incorporate distinct strategies to preserve nondominated solutions along the iterations. The performance of the multiobjective PSO (MOPSO) is usually evaluated by considering the resulting swarm at the end of the algorithm. In this paper, two indices based on the Shannon entropy are presented, to study the swarm dynamic evolution during the MOPSO execution. The results show that both indices are useful for analyzing the diversity and convergence of multiobjective algorithms.

## 1. Introduction

Multiobjective optimization (MOO) consists either in minimizing or in maximizing a set of objective functions subject to some constraints. In these problems, the objective functions are conflicting, leading to several vectors of decision variables. Each vector represents a possible solution that solves the problem with different trade-offs among the design objectives. Evolutionary and social-based algorithms have attracted the attention of many researchers, because they are frequently superior to conventional mathematical techniques due to their stochastic proprieties [1].

The MOO is inspired by biological phenomena and adopts a population that evolves during several generations. The PSO nature metaphor mimics the behavior of birds flocking or fish schooling [2]. Each bird or fish is represented by a particle with two components, namely by its position and velocity. A set of particles forms the swarm that evolves during several iterations giving rise to a powerful optimization method.

The particle swarm optimization’s (PSO) simplicity and success led to its application in problems where more than one optimization criterion is considered. Many techniques, such as those borrowed from genetic algorithms (GA) [3,4], have been developed to find a set of solutions belonging to the Pareto front. Since the multiobjective PSO (MOPSO) proposal [5], the algorithm was used in a wide range of applications [6,7], and a considerable number of variants of refined MOPSO were developed in order to improve its performance [8,9].

The performance of multiobjective algorithms is usually analyzed at the end of their execution, and its success is measured by means of several metrics proposed in the literature [10]. Additionally, in ambiguous situations, the use of nonparametric tests can be adopted [11,12]. Some of the proposed indices are based on Shannon entropy. Wang et al. [13] presented a method revealing interesting results: (i) the computational effort increases linearly with the number of solutions, (ii) the metric qualifies the combination of uniformity and coverage of the Pareto set, and (iii) it determines when the evolution has reached its maturity. LinLin and Yunfang [14] proposed a technique to measure the performance of multiobjective problems that not only indicates when the algorithm should be stopped, but can also compare the performance of multiobjective algorithms. The technique adopts entropy that is evaluated regarding the solution density in a gridded space.

Other studies tried to unravel the population dynamics during time evolution [15,16,17,18,19,20]. Farhang-Mehr and Azarm [15] developed an entropy-based metric to assess the diversity in a MOEA during the run time. To measure the entropy, a grid of cells was used, where solutions belonging to the same cell are considered identical. They orthogonalize and project them into a plane to count 3D nondominated solutions. Deb and Jain [16] proposed two multiobjective running metrics, one for measuring the convergence and the other for assessing the diversity among solutions. Myers and Hancock [17] suggested the use of the Shannon entropy to evaluate the run-time performance of a GA to solve labeling problems. The entropy measured in the parameter space is used to provide useful information about the algorithm state. Myers and Hancock concluded that populations with entropy smaller than a given threshold become saturated and their diversity disappears. Pires et al. [18] studied the signal propagation during the evolution of a GA. The mutation operator signal suffers a perturbation during some generations, and the corresponding fitness variation is analyzed. Pires et al. adopted the Shannon entropy to study the dynamics of MOPSO [19] and nonsorting GA II [20] during their execution. Wu et al. proposed a MOEA considering individual density (cell density) where the Shannon entropy was used to estimate the evolution state [21].

Taking these ideas in consideration, this paper studies the dynamics and self-organization of solutions during the MOPSO execution. The study analyzed two entropy indices considering three optimization functions with different swarm and archive sizes.

The main contributions of the paper are:

- New diversity indices inspired by physics and biologic systems.
- A good agreement of measures between the indices.
- Identification of stagnating states during the evolution.

Section 2 describes the method adopted in the work and includes a brief description of the main entropy concepts. Section 3 presents the indices for measuring the population diversity. Section 4 formulates the functions to be optimized and analyzes the simulations results. Finally, Section 5 outlines the main conclusions and the perspectives toward future work.

## 2. Methodology and Entropy Concepts

A careful look into MOPSO reveals a need to understand the dynamics during successive iterations with a particular focus on the particles’ convergence to the nondominated front and particle diversity. For this purpose, the Shannon entropy is used in the follow-up, and a set of tests is performed considering different optimization functions and archive sizes. Since the MOPSO algorithm is stochastic, a battery of tests is required to generate a representative statistical sample [22].

The entropy is associated with several concepts and interpretations [23]. Boltzmann used entropy to describe systems that evolve from ordered to disordered states. *Spreading* was used by Guggenheim to indicate the diffusion of an energy system from a small to a large volume. Lewis stated that in a spontaneous expansion gas in an isolated system, *information* regarding the particles’ locations decreases, while the missing information or *uncertainty* increases.

Shannon [24] developed a theory to quantify the information loss in the transmission of a given message. The study was carried out in a communication channel and focused on the physical and statistical constraints that limit the message transmission. Shannon defined entropy *H* as a measure of information, given by:

(1) $$H\left(X\right)=-K\sum_{x\in X}p_{i}\left(x\right)\log p_{i}\left(x\right).$$

This expression considers a discrete random variable x∈X characterized by the probability distribution p(x). The parameter *K* is a positive constant, often set to 1, and is used to express *H* in a given unit of measure.

The Shannon entropy can be easily extended to multidimensional random variables. For a pair of random variables (x,y)∈(X,Y), entropy is defined as:

(2) $$H\left(X,Y\right)=-K\sum_{x\in X}\sum_{y\in Y}p_{i}\left(x,y\right)\log p_{i}\left(x,y\right).$$

## 3. Entropy Indices for Assessing the MOPSO

In this section, two indices for measuring the entropy in a MOPSO are presented. The first one captures the particle diversity, while the second addresses the front diversity.

### 3.1. Particle Diversity

The index to measure the particle diversity was proposed previously [25]. The index follows a particular interpretation of entropy. Indeed, entropy can express the spreading of a system energy from a `better located’ state to a `more distributed’ one. Taking this idea in mind, the minimum spanning tree that connects all the archive particles, *A*, where each connection belongs to the set of edges that connects all the #A particles with the minimal edge distance, was considered. Let $d_{i}$ be one of these edges, where i∈{1,2,…,#A-1}, and $p_{i}$ is a probability given by the following Equation:

(3) $$p_{i}=\frac{d_{i}}{\sum_{j=1}^{\#A-1}d_{j}}.$$

The particle diversity index is based on this point of view and can be represented as:

(4) $$H\left(X\right)=-\sum_{i=1}^{\#A-1}p_{i}\log p_{i}.$$

### 3.2. Front Level Heterogeneity

In ecology, the diversity measure of different populations species is equated with the uncertainty that occurs when selecting randomly one individual species from the populations [26]. The information content, or population diversity, can be defined in several ways [27], and one of them is explained in the follow-up.

Consider a population with *s* species in proportion to $p_{i}=\frac{n_{i}}{\#A}$, i.e., $\left\{p_{1},p_{2},\ldots ,p_{s}\right\}$, where $n_{i}$ is the number of elements of the *i*th species and *s* denotes the total number of species. Then the population diversity is given by Shannon and Weaver’s formula [28]:

(5) $$H^{\prime }\left(X\right)=-\sum_{i=1}^{s}p_{i}\log p_{i}.$$

Taking this idea in mind, Expression (5) is used to measure the Shannon front level diversity, where $n_{i}$ and *s* are the number of particles in each front and the total number of fronts, respectively. This index is called front level heterogeneity in order to avoid confusion with the particle diversity index. In MOPSO, at later evolution stages, when only the nondominated front exists in the swarm, the entropy heterogeneity $H^{\prime }\left(X\right)$ is zero.

## 4. Simulations Results

This section presents the results obtained for 3 optimization problems $P_{i},i=1,2,3$, and different archive sizes. The dynamic behavior of algorithms was studied using the two proposed indices.

The optimization problems $P_{1}$ and $P_{2}$ (known as DTLZ2 and DTLZ4 [29]) are defined by Equations (6) and (7), and problem $P_{3}$ (known as UF8 in CEC 2009 special session competition [30]) is formulated in Equation (8) as follows:

(6) $$\left\{\begin{array}{ccc} \min P_{1} & = & \left[f_{11}\left(X\right),f_{12}\left(X\right),f_{13}\left(X\right)\right]\\ f_{11}\left(X\right) & = & \left[1+g_{1}\left(X\right)\right]\cos\left(x_{1}\pi /2\right)\cos\left(x_{2}\pi /2\right)\\ f_{12}\left(X\right) & = & \left[1+g_{1}\left(X\right)\right]\cos\left(x_{1}\pi /2\right)\sin\left(x_{2}\pi /2\right)\\ f_{13}\left(X\right) & = & \left[1+g_{1}\left(X\right)\right]\sin\left(x_{1}\pi /2\right)\\ g_{1}\left(X\right) & = & 1+9\sum_{i=3}^{m}{\left(x_{i}-0.5\right)}^{2} \end{array}\right.$$

(7) $$\left\{\begin{array}{ccc} \min P_{2} & = & \left[f_{21}\left(X\right),f_{22}\left(X\right),f_{23}\left(X\right)\right]\\ f_{21}\left(X\right) & = & \left[1+g_{2}\left(X\right)\right]\cos\left(x_{1}^{\alpha }\pi /2\right)\cos\left(x_{2}^{\alpha }\pi /2\right)\\ f_{22}\left(X\right) & = & \left[1+g_{2}\left(X\right)\right]\cos\left(x_{1}^{\alpha }\pi /2\right)\sin\left(x_{2}^{\alpha }\pi /2\right)\\ f_{23}\left(X\right) & = & \left[1+g_{2}\left(X\right)\right]\sin\left(x_{1}^{\alpha }\pi /2\right)\\ g_{2}\left(X\right) & = & 1+9\sum_{i=3}^{m}{\left(x_{i}^{\alpha }-0.5\right)}^{2} \end{array}\right.$$

(8) $$\left\{\begin{array}{ccc} \min P_{3} & = & \left[f_{31}\left(X\right),f_{32}\left(X\right),f_{33}\left(X\right)\right]\\ f_{31}\left(X\right) & = & \cos\left(0.5x_{1}\pi \right)\cos\left(0.5x_{2}\pi \right)+\frac{2}{|J_{1}|}\sum_{j\in J_{1}}g_{j}\left(X\right)\\ f_{32}\left(X\right) & = & \cos\left(0.5x_{1}\pi \right)\sin\left(0.5x_{2}\pi \right)+\frac{2}{|J_{2}|}\sum_{j\in J_{2}}g_{j}\left(X\right)\\ f_{33}\left(X\right) & = & \sin\left(0.5x_{1}\pi \right)+\frac{2}{|J_{3}|}\sum_{j\in J_{3}}g_{j}\left(X\right)\\ g_{j}\left(X\right) & = & {\left(x_{j}-2x_{2}\sin\left(2\pi x_{1}+\frac{j\pi }{m}\right)\right)}^{2}\\ J_{1} & = & \left\{j|3\leq j\leq m,\mathrm{and}j+2\mathrm{is}\mathrm{multiple}\mathrm{of}3\right\}\\ J_{2} & = & \left\{j|3\leq j\leq m,\mathrm{and}j+1\mathrm{is}\mathrm{multiple}\mathrm{of}3\right\}\\ J_{3} & = & \left\{j|3\leq j\leq m,\mathrm{and}j\mathrm{is}\mathrm{multiple}\mathrm{of}3\right\} \end{array}\right.$$

where *m* is the number of parameters, $f_{ij}$ is the objective j∈{1,2,3} of problem i∈{1,2,3}, and *g* represent some auxiliary functions in order to simplify the expressions. For $\left\{P_{1},P_{2}\right\}$, the parameter intervals are set to $x_{i}\in \left[0,1\right]$. In Equation (7), the parameter value α=100 allows a meta-variable mapping, $x_{i}\to x_{i}^{2}$, between the two functions [29]. For $\left\{P_{3}\right\}$, $x_{i}\in \left[0,1\right]$ if i≤2 and $x_{i}\in \left[-2,2\right]$ if 2<i≤m-2. For $\left\{P_{1},P_{2},P_{3}\right\}$, the number of parameters is set to m={12,12,30}.

These problems are to be optimized using a MOPSO [5,6], where the search is driven by a population of particles that move using the equations:

(9) $$v_{i}^{t+1}=w\cdot v_{i}^{t}+\phi_{1}\cdot \mathrm{rand}\left(0,1\right)\cdot \left(b_{i}-x_{i}^{t}\right)+\phi_{2}\cdot \mathrm{rand}\left(0,1\right)\cdot \left(g_{i}-x_{i}^{t}\right),$$

(10) $$x_{i}^{t+1}=x_{i}^{t}+v_{i}^{t+1},$$

where *t* is the iteration number, *w* denotes the inertia coefficient, and the positions $x_{i}$ and velocities $v_{i}$ are codified by means of real numbers. In order to start with an high exploration rate of the search space, *w* is initialized with the value 0.7 and decreases linearly with *t* to 0.25. In the stages where *w* is near the value 0.25, more importance is given to the local search rather then the global one. In the particle motion, the same influence is given to the local best particle position $b_{i}$ and the position of the `best’ particle $g_{i}$. Therefore, the cognitive and social coefficients are set to the values $\phi_{1}=\phi_{2}=0.8$. In a nondominated set, there is no best solution. Consequently, to choose a particle with similar characteristics while incorporating uncertainty, a particle determines its `global best’, or guide, by randomly selecting three particles from the archive and picking up the nearest particle $g_{i}$.

The archive is updated, at the end of each iteration, using a (μ+λ) strategy among the archive, #μ, and swarm, #λ, solutions. Therefore, the best μ solutions are chosen among the archive and population solutions. The solutions are selected according to the maximin sorting scheme [12].

Four swarm sizes with #N={250,300,350,400} particles and four archive sizes of #A={50,100,150,200} particles are adopted, resulting in a total of $4^{2}$ different experiments. For each experiment, 21 distinct runs were performed, their entropies evaluated, and the medians of the particles and the populations’ diversities and heterogeneities $H_{m}=\mathrm{median}\left(H\right)$ and $H_{m}^{\prime }=\mathrm{median}\left(H^{\prime }\right)$ at each iteration *t* taken as representing the entropy evolution at that instant.

This section presents the entropy evolution for those experiments addressing the problems $\{P_{1}$, $P_{2}$, $P_{3}\}$.

### 4.1. Results of DTLZ Problems Optimization

**Figure 1:**
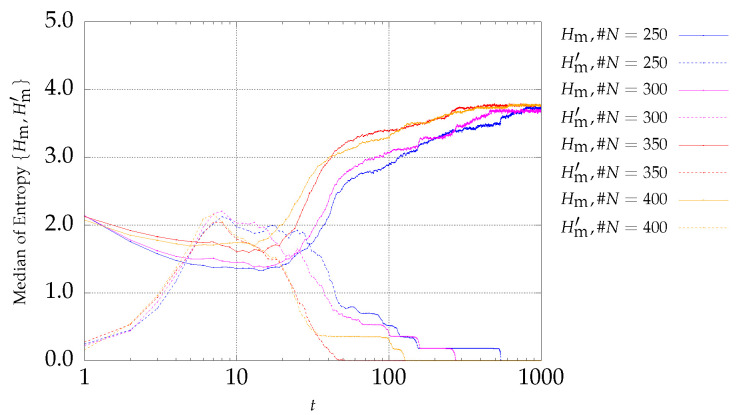
Evolution of $H_{m}$ (continuous lines) and $H_{m}^{\prime }$ (dotted lines) versus the iterations *t* of the MSPSO iterations for $P_{1}$ and #A=50.

**Figure 2:**
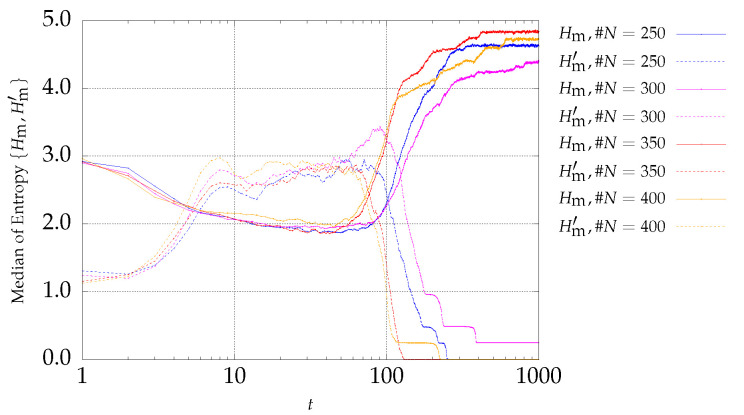
Evolution of $H_{m}$ (continuous lines) and $H_{m}^{\prime }$ (dotted lines) versus the iterations *t* of the MSPSO iterations for $P_{1}$ and #A=200.

The first optimization functions to be considered belong to problem $P_{1}$, with three objectives described by Equation (6). Expressions (4) and (5) are adopted to monitoring the MOPSO evolution. The results are depicted in Figure 1 and Figure 2 for archive sizes of $\#A_{1}=50$ and $\#A_{4}=200$ particles, respectively. The charts show the median entropies $H_{m}$ and $H_{m}^{\prime }$ versus the iteration *t*, for experiments with #N={250,300,350,400} particles. The curves with the `solid’ and `dotted’ lines represent $H_{m}$ and $H_{m}^{\prime }$, respectively.

It can be observed that the two entropy signals are inversely correlated. In what concerns $H_{m}^{\prime }$, it is verified that it starts with a low value and increases during the first iterations. Afterwards, $H_{m}^{\prime }$ remains almost stationary during some interactions and finally decreases to zero. This means that at the early stages, the number of fronts increases, remains constant during a certain number of iterations, and then decreases until only one front remains (i.e., the nondominated front), when $H_{m}^{\prime }=0$. As the archive size increases, the initial transient takes more iterations, because as the number of archive size increases, it gets more difficult to find a larger number of nondominated particles in the same period. On the other hand, since the number of particles is larger, it is possible for more fronts to emerge. Therefore, $H_{m}^{\prime }$ takes more iterations to approach zero as the archive size increases.

**Figure 3:**
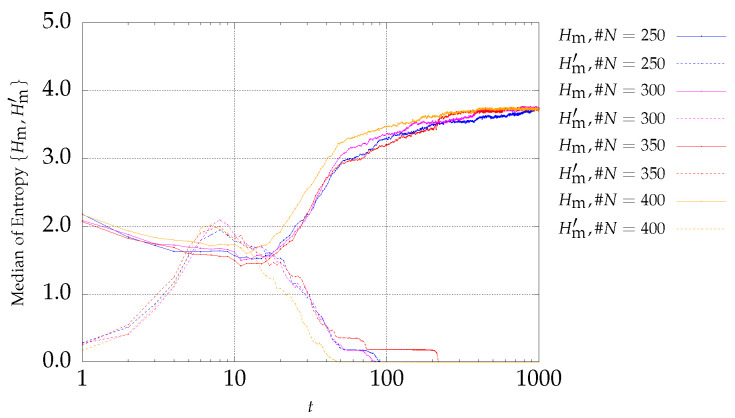
Evolution of $H_{m}$ (continuous lines) and $H_{m}^{\prime }$ (dotted lines) versus the iterations *t* of the MSPSO iterations for $P_{2}$ and #A=50.

**Figure 4:**
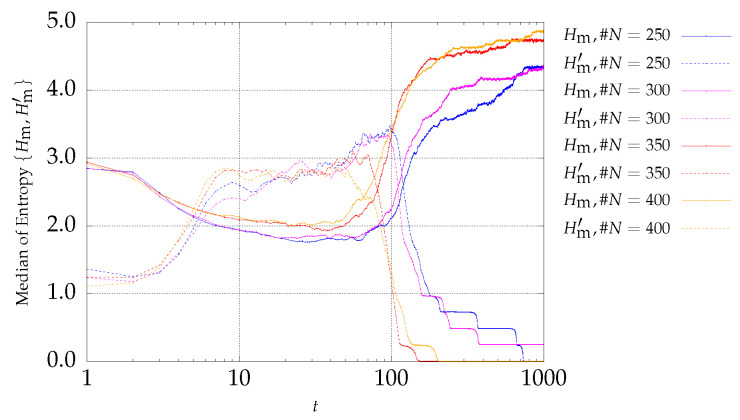
Evolution of $H_{m}$ (continuous lines) and $H_{m}^{\prime }$ (dotted lines) versus the iterations *t* of the MSPSO iterations for $P_{2}$ and #A=200.

Figure 3 and Figure 4 present the entropy indices for the $P_{2}$ problem. A behavior similar to the one exhibited by $P_{1}$ is visible.

### 4.2. Results of $P_{3}$ Optimization

**Figure 5:**
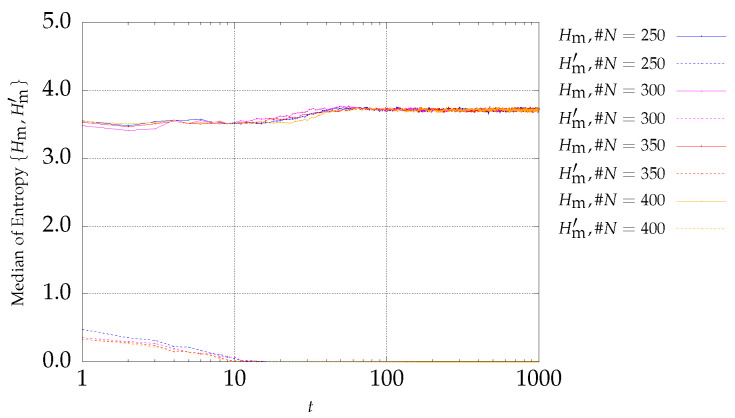
Evolution of $H_{m}$ (continuous lines) and $H_{m}^{\prime }$ (dotted lines) versus the iterations *t* of the MSPSO iterations for $P_{3}$ and #A=50.

**Figure 6:**
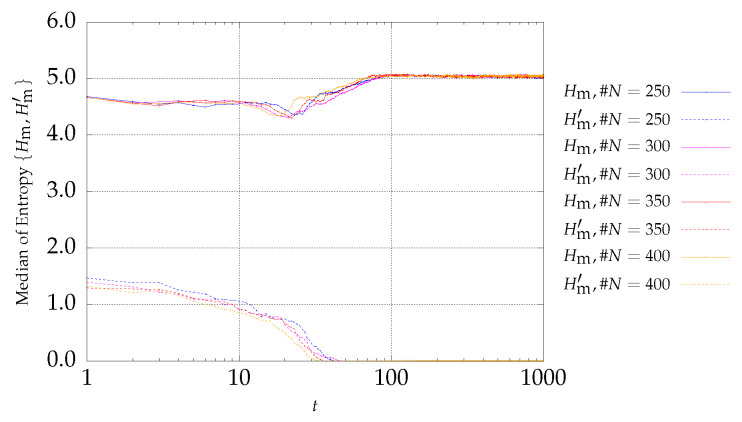
Evolution of $H_{m}$ (continuous lines) and $H_{m}^{\prime }$ (dotted lines) versus the iterations *t* of the MSPSO iterations for $P_{3}$ and #A=200.

Figure 5 and Figure 6 present the entropy indices for the $P_{3}$ problem. Here, $H_{m}^{\prime }$ starts by decreasing, showing that the number of fronts begins to reduce until only one front remains. Since the initial value of $H_{m}^{\prime }$ is low, the number of initial fronts is also small. On the other hand, the diversity between the particles begins with a high value and increases slightly during the iterations.

The number of fronts, i.e., the entropy front level diversity, can increase or decrease at early stages depending on the optimization problem.

### 4.3. Correlation Coefficient

The results reveal a correlation between $H_{m}$ and $H_{m}^{\prime }$. The Pearson correlation coefficient *r* measures the strength and direction of a relationship between two indices, during $T=10^{3}$ iterations:

(11) $$r=\frac{\#A\sum_{t=1}^{T}H_{m}\left(t\right)H_{m}^{\prime }\left(t\right)-\sum_{t=1}^{T}H_{m}\left(t\right)\sum_{t=1}^{T}H_{m}^{\prime }\left(t\right)}{\sqrt{\left[\#A\sum_{t=1}^{T}H_{m}^{2}\left(t\right)-{\left(\sum_{t=1}^{T}H_{m}\left(t\right)\right)}^{2}\right]\left[\#A\sum_{t=1}^{T}{H^{\prime }}_{m}^{2}\left(t\right)-{\left(\sum_{t=1}^{T}H_{m}^{\prime }\left(t\right)\right)}^{2}\right]}}.$$

Table 1 presents the correlation between the particle diversity and front heterogeneity entropies. Column 1 indicates the archive size, column 2 stands for the swarm size, and the symbols $r_{1}$, $r_{2}$, and $r_{3}$ represent the correlation between $H_{m}$ and $H_{m}^{\prime }$ for the problems $P_{1}$, $P_{2}$, and $P_{3}$, respectively. An almost perfect negative relationship can be observed between them for each DTLZ problem. The relationship for the $P_{3}$ problem signals is moderate for the archive size of #A=50 particles, but it is stronger for the other archive sizes. These values of *r* demonstrate that the 2 indices are in good agreement, concluding that the diversity solution is highly correlated in the number of fronts.

### 4.4. Archive Evolution

**Figure 7:**
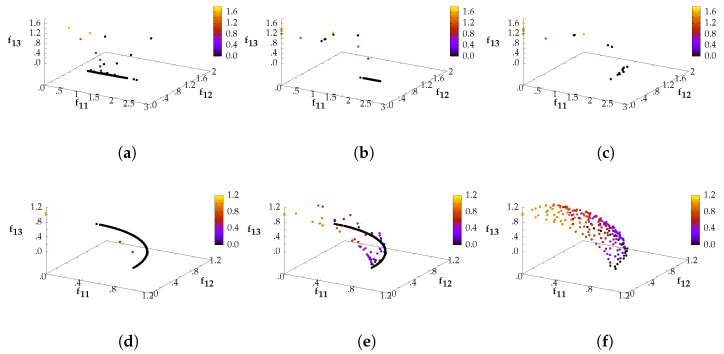
MPSO evolution for an isolated run of the *P*_2_ problem with #*N* = 250 and #*A* = 200 for iteration (**a**) *t* = 1, (**b**) *t* = 4, (**c**) *t* = 80, (**d**) *t* = 110, (**e**) *t* = 400 and (**f**) *t* = 1000.

In order to show the particle distribution of the archive, the position of the particles archive is plotted at t={0,3,80,110,400,1000} iterations, for problem $P_{2}$, with #N=250 and #A=200. The iterations are chosen at different stages of the evolution, namely, at the beginning (t=1), when the diversity index drops down (t=4), at the end of this stagnation phase (t=80), after an abrupt increase of the index (t=110), after some iterations (t=400), and at the end of the run (t=1000). The plots represented in Figure 7 illustrate the result for one single run.

It can be observed that the entropy achieves the maximum value when the archive solutions are well dispersed.

## 5. Conclusions and Future Work

Two indices based on entropy were proposed to characterize the MOPSO dynamics. This work measures the diversity of the archive during the evolution, adopting one possible interpretation of entropy. The first index, the particle diversity, is used to measure the archive diversity between particles. The second index, borrowed from ecology, measures the species heterogeneity, in this case the front level heterogeneity. The indices were evaluated using different approaches, but both entropy indices were in good agreement, revealing that solution diversity is correlated with the number of fronts. The particle diversity indices when stagnated reveal that the algorithm has converged. On the other hand, the front level heterogeneity, when reaching zero, indicates that there is only one front in the archive.

For most MOPSO reported in the literature, the performance evaluation is analyzed at the end of the algorithm, by comparing the final front extension, spreading, and diversity. The indices formulated in this paper can be used to analyze the convergence rate during the time evolution. In future work, the indices will be used to identify stained stages of MOPSO and introduce mechanisms to promote the dispersion of particles during evolution optimization.

## Figures and Tables

**Table 1:** Pearson correlation coefficient *r* between $H_{m}$ and $H_{m}^{\prime }$.

Archive Size ($A_{p}$)	Swarm Size ($N_{p}$)	$r_{1}$	$r_{2}$	$r_{3}$
50	250	-0.97	-0.92	-0.51
	300	-0.97	-0.92	-0.66
	350	-0.88	-0.96	-0.50
	400	-0.93	-0.89	-0.43
100	250	-0.99	-0.99	-0.81
	300	-0.99	-0.92	-0.69
	350	-0.97	-0.96	-0.73
	400	-0.96	-0.95	-0.75
150	250	-0.98	-0.98	-0.82
	300	-0.98	-0.99	-0.74
	350	-0.97	-0.99	-0.80
	400	-0.98	-0.97	-0.69
200	250	-0.99	-0.98	-0.81
	300	-0.98	-0.98	-0.72
	350	-0.97	-0.98	-0.75
	400	-0.96	-0.98	-0.77
